# Pectin Influences the Absorption and Metabolism of Polyphenols from Blackcurrant and Green Tea in Rats

**DOI:** 10.3390/foods10040813

**Published:** 2021-04-09

**Authors:** Gunaranjan Paturi, Christine A. Butts, Nigel I. Joyce, Paula E. Rippon, Sarah C. Morrison, Duncan I. Hedderley, Carolyn E. Lister

**Affiliations:** 1 The New Zealand Institute for Plant and Food Research Limited, Private Bag 92169, Auckland 1142, New Zealand; 2 The New Zealand Institute for Plant and Food Research Limited, Private Bag 11600, Palmerston North 4442, New Zealand; duncan.hedderley@plantandfood.co.nz; 3 The New Zealand Institute for Plant and Food Research Limited, Private Bag 4704, Christchurch 8140, New Zealand; nigel.joyce@plantandfood.co.nz (N.I.J.); paula.rippon@plantandfood.co.nz (P.E.R.); sarah.morrison@plantandfood.co.nz (S.C.M.); carolyn.lister@plantandfood.co.nz (C.E.L.)

**Keywords:** dietary fiber, functional foods, metabolites, plant-based foods

## Abstract

Consumption of polyphenols and dietary fiber as part of a normal diet is beneficial to human health. In this study, we examined whether different amounts of dietary soluble fiber (pectin) affect the absorption and metabolism of polyphenols from blackcurrant and green tea in rats. After 28 days, the rats fed blackcurrant and green tea with pectin (4 or 8%) had significantly lower body weight gain and food intake compared to the rats fed a control diet. Rats fed a blackcurrant and green tea diet with 8% pectin had significantly higher fecal nitrogen output and lower protein digestibility. No polyphenols were observed in the urine, feces and plasma of rats fed the control diet. Parent catechins and flavonols were absent in urine obtained from all diet groups. Gallocatechin glucuronide was only observed in the plasma of rats fed the blackcurrant and green tea diet without pectin. Meanwhile, epicatechin and catechin gallate were present in the feces of rats fed a blackcurrant and green tea diet with and without 4% pectin. Pectin (4 or 8%) added to the blackcurrant and green tea diet increased the plasma antioxidant capacity in rats. Inclusion of pectin in the diet altered the host absorption and metabolism of polyphenols from blackcurrant and green tea.

## 1. Introduction

Polyphenols found in fruit and vegetables offer numerous health benefits, including protection against cardiovascular disease [1]. The biological activity of polyphenolic compounds has been attributed to their antioxidant properties due to free radical scavenging activity, metal chelating properties and enzymatic activity. The bioavailability of polyphenols is highly variable and dependent on factors such as the structure and conjugation of the polyphenol, food matrix components and interactions with the gastrointestinal system [2,3]. The in vivo effect of a compound depends on its absorption and elimination kinetics, the nature and extent of its metabolism and the activity of circulating compounds. Flavonoids are a diverse group of polyphenols found naturally in several fruits and vegetables. Flavonoid metabolism occurs in the small intestine and liver, whereas the unabsorbed flavonoids undergo microbiota-mediated ring-fission, producing phenolic acids that are absorbed and excreted in the urine [4].

The health benefits of polyphenols have been associated with the parent compounds in food, but it may in fact be the presence of their metabolites in the blood and tissues that confers these biological properties [3,5]. Polyphenols can directly influence the cellular functions and indirectly through the gut microbiota [6]. Approximately 5–10% of total polyphenols are absorbed in the small intestine, and the remaining polyphenols travel to the large intestine, where they are either metabolized by the resident bacteria or excreted in the feces. Dietary fiber is essential for optimal health due to its physicochemical properties that enhance gut health and overall wellness [7]. In an earlier study in rats fed a diet with fermentable fiber and blackcurrant, we observed synergistic health benefits greater than those of the individual components [8]. A healthy diet comprises a mix of macronutrients and micronutrients, resulting in a complex food matrix that can affect the bioaccessibility of polyphenols in the gut [9,10]. Previous studies suggest that dietary fiber plays an important role in controlling the amount of polyphenols accessible in the upper and lower regions of the gut [11]. Blackcurrants are rich in anthocyanins while green tea is rich in catechins. In vivo effects of dietary fiber on the bioavailability of polyphenols from blackcurrant and green tea remain largely unknown. Understanding the association between dietary fiber and polyphenols when consumed as part of a diet can give new insights into the complex processes that occur in the gut and, most importantly, this information can be used to inform food choices, with positive health benefits. In the present study, we investigated the effects of soluble fiber (pectin) on the absorption and metabolism of dietary nitrogen and the polyphenols from blackcurrant and green tea in rats. Plasma antioxidant capacity, corticosterone and uric acid were also examined in rats fed the experimental diets.

## 2. Materials and Methods

### 2.1. Animal Experiment

This study was carried out with approval from AgResearch Grasslands Animal Ethics Committee (Palmerston North) according to the Animal Welfare Act 1999, New Zealand. Animal trial was carried out in a temperature-controlled room (22 ± 1 °C, humidity of 60 ± 5%) with a 12 h light/dark cycle. Male Sprague-Dawley rats were raised in family groups and fed a commercial diet post-weaning. Six-week-old rats were then transferred to individual hanging cages and fed the control diet *ad libitum* for 1 week. Rats were randomly allocated to experimental diets and fed for 28 days (*n* = 10 per diet). The experimental diets included a control diet and three diets that contained blackcurrant extract (4%) and green tea extract (0.02%) with different concentrations of pectin (0, 4 or 8%) (Table 1).

Rat body weight, food intake and fecal output were recorded weekly during the experiment. In the last 7-day period, rats were transferred to metabolism cages and total feces and urine output were collected daily and stored at −20 °C until prepared for analysis. Feces were freeze-dried, sieved to remove any spilled diet and finely ground. Urine was collected in containers with 0.5 mL 1M HCl. At the end of 28 days, the rats were euthanized via CO_2_ asphyxiation. Blood samples were taken via cardiac puncture and stored on ice until plasma was prepared by centrifugation at 2000× *g* for 10 min at 4 °C. The plasma was stored in aliquots for corticosterone, metabolite, uric acid and plasma antioxidant capacity analyses.

### 2.2. Nitrogen and Corticosterone

Total nitrogen content in the rat diets, feces and urine were determined by combustion method (AOAC 1990) using a LECO FP-2000 analyzer (Leco Corp., St. Joseph, MI, USA). Corticosterone concentrations in plasma were determined using a double antibody radioimmunoassay kit (RIA DSL-80100, Diagnostic Systems Laboratories, Webster, TX, USA). All the samples were measured in duplicate and the variation between duplicate samples was <10%.

### 2.3. Preparation of Samples for Polyphenol Analysis

Experimental diets were extracted in 85% aqueous methanol, vortexed, centrifuged, and the supernatant filtered through 0.22 µm nylon filter prior to liquid chromatography–mass spectrometry (LC–MS) analysis. Urine, feces and plasma samples were extracted according to the method described by Mullen et al. [12] and Gee et al. [13]. Briefly, urine was centrifuged and acidified (1/60 volume of 12 M HCl) to stabilize the flavonoids and stored at −80 °C until analysis. Fecal samples were freeze-dried, finely ground and extracted on ice with 2.5 mL of 70% aqueous methanol containing 1% sodium ascorbate. The samples were thoroughly mixed, centrifuged (3220× *g*, 10 min, 4 °C) and supernatants removed. The supernatants were then evaporated to dryness *in vacuo* (<45 °C), re-dissolved in methanol (1 mL) and filtered (0.22 µm polytetrafluoroethylene) prior to LC–MS analysis. For plasma samples, acetonitrile (2.5 vol) was used to precipitate the plasma proteins and extract flavonoid metabolites. Formic acid (50% *v*/*v*, 0.03 vol) was added to acidify plasma, and ascorbic acid (10 mM, 0.1 vol) was added to help stabilize the samples during processing. Plasma (1 vol) was added dropwise, and the samples were vortexed for 30 s every 2 min over a 10 min period before centrifugation (2800× *g*, 20 min, 4 °C). Supernatants were collected and evaporated to dryness *in vacuo* (<45 °C) and then re-suspended in 1% formic acid (0.9 vol) and methanol (0.1 vol). The samples were stored at −80 °C for metabolite analysis.

### 2.4. Liquid Chromatography–Mass Spectrometry

The LC–MS system consisted of a Thermo Electron Corporation (San Jose, CA, USA) Finnigan Surveyor MS pump, Finnigan MicroAS auto-sampler, Finnigan Surveyor PDA detector and a ThermaSphere TS-130 column heater (Phenomenex, Torrance, CA, USA). Sample vials were placed in a cooled auto-sampler rack maintained at 10 °C. Urine, feces and plasma sample volumes injected were 5 µL. Each sample was separated with a mobile phase consisting of 0.1% formic acid in water and 0.1% formic acid in acetonitrile by reverse-phase chromatography (Aqua guard cartridge 4 × 2 mm, 10 μm and Synergi Hydro-RP C18, 4 μm, 80 Å, 250 × 2.1 mm, Phenomenex, Torrance, CA, USA) maintained at 30 °C with a flow rate of 200 μL/min. The eluent was scanned by PDA (190–600 nm) and API-MS (LTQ, 2D linear ion-trap, Thermo-Finnigan) with electrospray ionization in the negative and positive mode. Data were acquired for precursor masses from *m*/*z* 120 to 2000 amu that were selected for MS^2^ fragmentation; subsequently, the two most abundant product ions were selected for MS^3^ and likewise MS^4^ based on the two most abundant ions from MS^3^. Data were processed with Xcalibur 2.05 software (Thermo Electron Corporation).

### 2.5. Total Peroxyl Radical-Trapping Antioxidant Parameter (TRAP) and Uric Acid

Plasma antioxidant capacity was determined by the method described by Valkonen and Kuusi [14] with some modifications. Dichlorofluorescein was oxidized by radicals that were generated by thermal decomposition of 2,2′-azobis (2-amidinopropane) dihydrochloride (AAPH) in an aqueous medium. The appearance of oxidation product, dichlorofluorescein, was monitored spectrophotometrically over time at 504 nm. The extent to which plasma could scavenge AAPH peroxyl radicals and prevent oxidation was measured as the lag phase. The TRAP value was expressed as µmoles peroxyl radicals trapped per liter of plasma using a known concentration of Trolox (water-soluble Vitamin E analogue) as an internal standard. Two aliquots of plasma from each rat were analyzed in separate microplate assays in triplicate, along with the control plasma. Further aliquots were analyzed as necessary when the variation between duplicate samples was >10%. Uric acid content in the rat plasma samples was analyzed by a modified Trinder method using the Aeroset c8000 system (Abbott, Chicago, IL, USA).

### 2.6. Statistical Analysis

Data were analyzed using analysis of variance (ANOVA) with diet as a factor. Tukey’s honestly significant difference (HSD at *p* = 0.05) was used to compare means when the ANOVA was significant. The TRAP values can be affected by the degree of hemolysis of the sample so this was included in the analysis. There were also likely to be differences between individual rats and between the microplates, which could increase the random variability in the data. Therefore, a mixed-model analysis with rats and plates as random effects and diet and hemolysis as fixed effects was performed on TRAP measurements. All the analyses were carried out using GenStat 18th edition (VSN International Ltd., Hemel Hempstead, UK).

## 3. Results and Discussion

### 3.1. Rat Food Intake, Body Weight and Fecal Nitrogen

Rats fed the blackcurrant and green tea diet with pectin (8%) had significantly lower food intake compared to those on other diets (*p* < 0.001) (Table 2). After 28 days, rat final body weights were similar between the diet groups (Table 2). However, rats fed blackcurrant and green tea diets gained less body weight than those rats fed the control diet. The reduction in rat food intake and weight gain with blackcurrant and green tea is likely due to the astringent or bitter properties of the polyphenols. The precipitation of salivary proteins on the tongue by polyphenols results in a feeling of constriction, roughness and dryness on the palate, therefore reducing the palatability of the diet to the rats [15]. Additionally, non-digestible carbohydrates and polyphenols can influence the neuropeptides involved in satiety [16]. Although the underlying mechanisms remain unknown in the current study, combining a blackcurrant and green tea diet with high soluble fiber (8% pectin) was effective in reducing rat food intake.

Protein intake was lower in rats fed a blackcurrant and green tea diet (*p* = 0.021) (Table 3). Rat fecal nitrogen outputs were significantly higher in blackcurrant and green tea diets with pectin (4 or 8%) when compared to the control diet (*p* < 0.001) (Table 3). The apparent nitrogen balance and protein digestibility measures were significantly different between the experimental diet groups (*p* < 0.001). Rats fed a blackcurrant and green tea diet with 8% pectin had high fecal nitrogen output and low protein digestibility, indicating that polyphenols may have bound to the dietary and endogenous proteins such as the digestive proteases, affecting protease activity and subsequently protein digestibility in the gut [17].

### 3.2. Polyphenols in Urine, Feces and Plasma of Rats

The absorption and metabolism of polyphenols was determined by the presence of polyphenol parent components and their metabolites in biological samples (urine, feces and plasma) from rats fed blackcurrant and green tea diets with or without pectin (Table 4). Relative peak areas of polyphenols detected in rat urine, feces and plasma are presented in Supplementary Tables S1–S3.

There were no catechins, anthocyanins, flavonols or phenolic acids detected in the control diet and those rats fed the control diet. For the rats given the blackcurrant and green tea diets, a total of eight catechins, five anthocyanins and four flavonols were identified.

**Figure 1:**
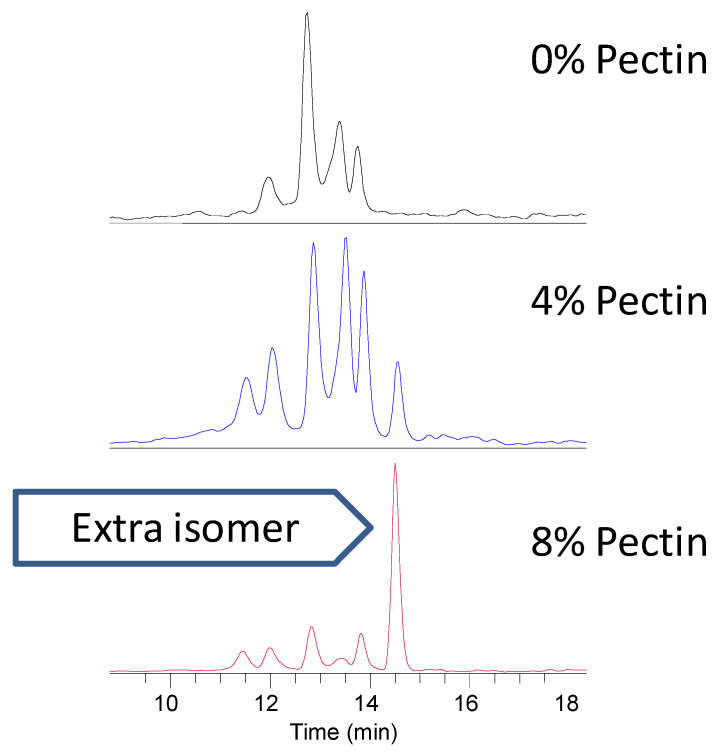
Increased methylation and additional isomers of methyl gallocatechin glucuronide identified in the urine of rats fed blackcurrant and green tea diet with 4 or 8% pectin.

Parent catechins and flavonols were not detected in the urine obtained from rats fed the blackcurrant and green tea diets, but there were parent anthocyanins present. Catechin, anthocyanin and flavonol metabolites were present in the rat urine. Adding pectin to the blackcurrant and green tea diet affected the digestion and absorption of catechins, as shown by their presence or absence in the urine. The inclusion of soluble fiber in the diet may have promoted further metabolism via methylation, as shown by the additional methyl gallocatechin glucuronide isomer and variable isomer profiles with the addition of 4 and 8% pectin to the blackcurrant and green tea diets (Figure 1). In the feces, catechins were only present as the parent compounds, while for the anthocyanins, there were both parent compounds and metabolites. Catechin gallate and epicatechin were not found in the feces of rats fed blackcurrant and green tea with 8% pectin. Petunidin and peonidin rutinosides were only found in the feces of rats that consumed blackcurrant and green tea without pectin. Two phenolic acids (3, 4 and 2, 4-dihydroxybenzoic acid) were present in the feces and urine of rats fed blackcurrant and green tea diets with and without pectin, but absent in the blood plasma. In the plasma, we found no anthocyanin parent compounds or their metabolites in rats fed blackcurrant and green tea diets. The only catechin parent compound present in the plasma was catechin. Gallocatechin glucuronide was present in the plasma of rats fed a blackcurrant and green tea diet without pectin but was absent in rats fed the diets supplemented with 4 and 8% pectin. The only flavonol parent compound present in the plasma was quercetin rutinoside.

Catechins are one of the important polyphenols found in tea and include an abundance of epigallocatechin gallate and epigallocatechin. Previous studies in animals and humans have shown that catechins have low absorption rates and therefore reduced bioavailability in blood circulation [18]. When substantial quantities of catechins pass from the small intestine to the large intestine, they can be catabolized by the bacteria to produce phenolic acids before being excreted in the feces. This could be the reason for the absence of epicatechin and catechin gallate in the feces of rats that consumed a blackcurrant and green tea diet containing 8% pectin. Similarly, there were some anthocyanins detected in the urine but not the feces of rats fed the diets containing pectin (4 or 8%). The anthocyanin-rich blackcurrant extract in the current study contained mainly delphinidin-3-glucoside, delphinidin-3-rutinoside, cyanidin-3-glucoside and cyanidin-3-rutinoside [8]. With some exceptions, anthocyanins appeared to be largely absorbed into the blood circulation, removed by the kidneys and consequently detected in the rat urine.

Inclusion of dietary fiber in a blackcurrant and green tea diet offers potential health benefits. This is evident from the observed presence and absence of polyphenols observed in the urine, feces and plasma of these rats. The addition of pectin, a fermentable fiber, affected the anthocyanin, catechin and flavonol metabolism, as indicated by the absence of some of the parent compounds and their metabolites in the urine, feces and plasma of rats fed diet with 4 and 8% pectin. Most polyphenols display limited bioavailability, but adding pectin to the diet can improve the polyphenol absorption by delaying the gastric emptying, which extends absorption in the small intestine, therefore enhancing the bioavailability of polyphenols [19,20,21]. Polyphenols that escape digestion in the small intestine reach the large intestine, where they can act as a nutrient source to some resident microorganisms, thus inducing changes in the microbiota composition. There is growing evidence in the literature that polyphenols are extensively metabolized and further converted by the gut microbiota into bioactive molecules that are absorbed through the gut barrier, entering blood circulation and peripheral organs, contributing to host physiological functions [16,22]. In previous studies, we found that blackcurrant and pectin altered the microbiota composition and increased short-chain fatty acids (SCFAs) concentrations in the gut, which can impact overall health and wellbeing [8,23].

It is worth mentioning that food source (cultivar, growing environment), chemical structure, conjugation, food matrix, food processing conditions, interactions with other compounds and host physiology can influence the bioavailability of polyphenols [3]. Future studies should investigate the role of the gut microbiota in polyphenol transformation and their contributions to health promotion. It also remains unknown how the polyphenol absorption and metabolism are affected by the formation of chemical complexes and colloidal structures between the polyphenols and dietary fiber within the food matrix and during the processes of digestion and absorption.

### 3.3. Plasma Corticosterone, TRAP and Uric Acid

There was no statistically significant difference in corticosterone concentrations between the diet groups, but there was a marked decrease in corticosterone concentrations in the rats fed blackcurrant and green tea diets containing pectin (4% or 8%) (Table 5). Polyphenols and non-digestible carbohydrate components in the diet can influence corticosterone levels. An earlier study in rats has reported the normalization of corticosterone concentrations by polyphenols [24]. Similarly, saccharolytic fermentation of dietary fiber occurs in the gut, releasing SCFAs that can also lower corticosterone in blood plasma [25].

Plasma TRAP concentrations tended to be higher in rats fed blackcurrant and green tea diets with either 4 or 8% pectin compared to the control diet (*p* = 0.046) (Table 6). The TRAP assay has been widely used to determine the antioxidant potential of food constituents [26]. Amongst fruit, high antioxidant capacity was found in berries, including blackcurrants [27,28]. In the current study, the combination of phenolic-rich plant extracts and pectin enhanced the plasma antioxidant capacity in rats. Further research in humans is needed to substantiate the health-promoting properties of the dietary combination of blackcurrant, green tea and pectin.

Endogenous plasma antioxidants are responsible for the homeostatic regulation of antioxidant status in blood. Uric acid is thought to contribute the greatest effect on TRAP [29]. In the present study, there was no correlation between TRAP and uric acid measurements (R^2^ = 0.05, *p* = 0.756). Plasma uric acid concentrations did not differ significantly between the diet groups (Table 6). However, rats that consumed a diet with 4 or 8% pectin tended to have lower uric acid, highlighting the beneficial role of dietary fiber in suppressing uric acid concentrations [30].

## 4. Conclusions

Blackcurrant and green tea diets with and without pectin showed differences in polyphenol absorption and metabolism in rats. Adding pectin to a blackcurrant and green tea diet decreased rat body weight gain and food intake and increased plasma antioxidant capacity. This study offers new knowledge of dietary combinations to deliver total polyphenols or specific metabolites of interest in vivo to promote health.

## Figures and Tables

**Table 1:** Ingredient compositions of experimental diets (g/kg).

	Control	Blackcurrant and Green Tea	Blackcurrant, Green Tea and Pectin (4%)	Blackcurrant, Green Tea and Pectin (8%)
Lactic casein ^1^	299.4	299.4	299.4	299.4
Vitamin mix ^2^	50	50	50	50
Mineral mix ^3^	50	50	50	50
Corn oil ^4^	97.6	97.6	97.6	97.6
Starch ^5^	413	372.8	332.8	292.8
Sucrose ^6^	40	40	40	40
Cellulose ^7^	50	50	50	50
Blackcurrant extract ^8^		40	40	40
Green tea extract ^9^		0.2	0.2	0.2
Pectin ^10^			40	80

^1^ Acid casein, New Zealand Milk Products Ltd., Wellington, New Zealand. ^2^ Mixture contains the following components: (mg/kg diet)—retinol acetate 5, DL-α-tocopheryl acetate 100, menadione 3, thiamin hydrochloride 5, riboflavin 7, pyridoxine hydrochloride 8, D-pantothenic acid 20, folic acid 2, nicotinic acid 20, D-biotin 1, myo-inositol 200, choline chloride 1500; (μg/kg diet)—ergocalciferol 25, cyanocobalamin 50. ^3^ Mixture contains the following components: (g/kg diet)—Ca 6.29, Cl 7.79, Mg 1.06, P 4.86, K 5.24, Na 1.97; (mg/kg diet)—Cr 1.97, Cu 10.7, Fe 424, Mn 78, Zn 48.2; (μg/kg diet)—Co 29, I 151, Mo 152, Se 151. ^4^ Essenté, Davis Trading, Palmerston North, New Zealand. ^5^ Wheaten cornflour, Golden Harvest, Primary Foods Ltd., Auckland, New Zealand. ^6^ Caster sugar, Chelsea, New Zealand Sugar Company Ltd., Auckland, New Zealand. ^7^ Avicel PH102, Commercial Minerals Ltd., Auckland, New Zealand. ^8^ Currantex 30, Just the Berries Ltd., Palmerston North, New Zealand. ^9^ Teavigo, DSM Nutritional Products Ltd., Heerlen, The Netherlands. ^10^ Pectin from citrus peel, Sigma-Aldrich, Auckland, New Zealand.

**Table 2:** Rat body weight and food intake.

	Final Body Weight (g)	Body Weight Gain (g)	Food Intake (g/day)
Control	309 ^a^	59 ^a^	16 ^b^
Blackcurrant and green tea	303 ^a^	41 ^b^	12 ^a,b^
Blackcurrant, green tea and pectin (4%)	316 ^a^	44 ^a,b^	17 ^b^
Blackcurrant, green tea and pectin (8%)	317 ^a^	50 ^a,b^	11 ^a^
Tukey HSD	30	16	4
*p* value	0.552	0.025	< 0.001

Mean values within the same column with a different letter differ significantly.

**Table 3:** Protein intake, fecal nitrogen and protein digestibility of rats fed the experimental diets.

	Protein Intake (g)	Protein Efficiency Ratio ^1^	Fecal Nitrogen Output (mg/5 Days)	Apparent Nitrogen Balance (mg/5 Days) ^2^	Protein Digestibility (%) ^3^
Control	42.8 ^a^	1.4 ^a^	202 ^c^	152 ^c^	78 ^a^
Blackcurrant and green tea	38.3 ^b^	1.0 ^a^	244 ^c^	220 ^b,c^	76 ^a^
Blackcurrant, green tea and pectin (4%)	40.6 ^a,b^	1.1 ^a^	307 ^b^	554 ^a^	78 ^a^
Blackcurrant, green tea and pectin (8%)	41.0 ^a,b^	1.2 ^a^	368 ^a^	356 ^b^	70 ^b^
Tukey HSD	3.7	0.4	56	170	4
*p* value	0.021	0.089	<0.001	<0.001	<0.001

Mean values within the same column with a different letter differ significantly. ^1^ Protein efficiency ratio = protein intake/weight gain. ^2^ Apparent nitrogen balance = nitrogen intake—(fecal nitrogen + urinary nitrogen). ^3^ Protein digestibility = nitrogen intake—(fecal nitrogen/nitrogen intake) × 100.

**Table 4:** Polyphenols identified in diets, and urine, feces and plasma of rats fed experimental diets.

	Diets ^1^	Urine			Feces			Plasma		
		Pectin (%)			Pectin (%)			Pectin (%)		
		0	4	8	0	4	8	0	4	8
Catechins										
Catechin	●							●	●	●
Epicatechin	●				●	●				
Gallocatechin	●				●	●	●			
Epigallocatechin	●				●	●	●			
Catechin gallate	●				●	●				
Epicatechin gallate	●				●	●	●			
Gallocatechin gallate	●				●	●	●			
Epigallocatechin gallate	●				●	●	●			
Catechin diglucuronide sulfate		●	●							
Epicatechin glucuronide		●	●	●				●	●	●
Methyl epicatechin glucuronide		●	●	●				●	●	●
Gallocatechin glucuronide		●	●	●				●		
Methyl gallocatechin glucuronide		●	●	●				●	●	●
Trimethyl catechin diglucuronide		●	●	●				●	●	●
Anthocyanins										
Cyanidin glucoside	●	●	●	●	●	●	●			
Cyanidin rutinoside	●	●	●	●	●	●	●			
Delphinidin glucoside	●	●	●							
Delphinidin rutinoside	●	●	●	●	●	●	●			
Petunidin glucoside		●	●	●	●	●				
Petunidin rutinoside		●	●	●	●					
Peonidin rutinoside	●	●	●	●	●					
Methyl cyanidin glucuronide		●	●							
Methyl delphinidin diglucuronide		●	●	●						
Methyl delphinidin glucuronide		●	●							
Flavonols										
Quercetin glucoside	●				●	●	●			
Quercetin rutinoside	●							●	●	●
Myricetin glucoside	●				●	●	●			
Myricetin rutinoside	●									
Quercetin glucuronide								●	●	●
Methyl quercetin glucuronide								●	●	●
Quercetin diglucuronide		●	●	●				●	●	●
Methyl quercetin diglucuronide		●	●	●				●	●	●
Myricetin glucuronide		●	●					●	●	●
Myricetin diglucuronide		●	●	●				●	●	●
Quercetin triglucuronide		●	●							
Kaempferol diglucuronide		●	●	●						
Myricetin triglucuronide		●	●	●						
Phenolic acids										
3,4 dihydroxybenzoic acid		●	●	●	●	●	●			
2,4 dihydroxybenzoic acid		●	●	●	●	●	●			

^1^ Blackcurrant and green tea diets with 0, 4 or 8% pectin. Polyphenols were not detected in control diet and rats fed the control diet. Dot (●) indicates the presence of a polyphenol.

**Table 5:** Rat plasma corticosterone.

	Corticosterone (ng/mL)
Control	69 ^a^
Blackcurrant and green tea	58 ^a^
Blackcurrant, green tea and pectin (4%)	48 ^a^
Blackcurrant, green tea and pectin (8%)	40 ^a^
Tukey HSD ^1^	713%
*p* value	0.369

^1^ Tukey HSD on the log scale, back-transformed. Two means are significantly different if the ratio of larger to smaller is greater than 7.13. Mean values within the same column with a different letter differ significantly.

**Table 6:** Total peroxyl radical-trapping antioxidant parameter (TRAP) and uric acid concentrations in the plasma of rats.

	TRAP (µmol/L)	Uric Acid (µmol/L)
Control	1085 ^a^	150 ^a^
Blackcurrant and green tea	1139 ^a^	148 ^a^
Blackcurrant, green tea and pectin (4%)	1189 ^a^	120 ^a^
Blackcurrant, green tea and pectin (8%)	1189 ^a^	124 ^a^
Tukey HSD	109	52
*p* value	0.046	0.267

Mean values within the same column with a different letter differ significantly.

## Data Availability

The data presented in this study are available on request from the corresponding author.

## Supplementary Materials

The following are available online at https://www.mdpi.com/article/10.3390/foods10040813/s1, Table S1: Relative peak areas of polyphenols in rat urine; Table S2: Relative peak areas of polyphenols in rat feces; Table S3: Relative peak areas of polyphenols in rat plasma.

## References

[1] Serino A., Salazar G. (2019). Protective role of polyphenols against vascular inflammation, aging and cardiovascular disease. Nutrients.

[2] Karaś M., Jakubczyk A., Szymanowska U., Złotek U., Zielińska E. (2017). Digestion and bioavailability of bioactive phytochemicals. Int. J. Food Sci. Technol..

[3] Teng H., Chen L. (2019). Polyphenols and bioavailability: An update. Crit. Rev. Food Sci. Nutr..

[4] Luca S.V., Macovei I., Bujor A., Miron A., Skalicka-Woźniak K., Aprotosoaie A.C., Trifan A. (2020). Bioactivity of dietary polyphenols: The role of metabolites. Crit. Rev. Food Sci. Nutr..

[5] Eker M.E., Aaby K., Budic-Leto I., Rimac Brnčić S., El S.N., Karakaya S., Simsek S., Manach C., Wiczkowski W., de Pascual-Teresa S. (2020). A review of factors affecting anthocyanin bioavailability: Possible implications for the inter-individual variability. Foods.

[6] Lavefve L., Howard L.R., Carbonero F. (2020). Berry polyphenols metabolism and impact on human gut microbiota and health. Food Funct..

[7] Li Y.O., Komarek A.R. (2017). Dietary fibre basics: Health, nutrition, analysis, and applications. Food Qual. Saf..

[8] Paturi G., Butts C.A., Monro J.A., Hedderley D. (2018). Effects of blackcurrant and dietary fibers on large intestinal health biomarkers in rats. Plant Foods Hum. Nutr..

[9] Bohn T. (2014). Dietary factors affecting polyphenol bioavailability. Nutr. Rev..

[10] Aguilera J.M. (2019). The food matrix: Implications in processing, nutrition and health. Crit. Rev. Food Sci. Nutr..

[11] Jakobek L., Matić P. (2019). Non-covalent dietary fiber—Polyphenol interactions and their influence on polyphenol bioaccessibility. Trends Food Sci. Technol..

[12] Mullen W., Edwards C.A., Crozier A. (2006). Absorption, excretion and metabolite profiling of methyl-, glucuronyl-, glucosyl- and sulpho-conjugates of quercetin in human plasma and urine after ingestion of onions. Br. J. Nutr..

[13] Gee J.M., Wroblewska M.A., Bennett R.N., Mellon F.A., Johnson I.T. (2004). Absorption and twenty-four-hour metabolism time-course of quercetin-3-*O*-glucoside in rats, in vivo. J. Sci. Food Agric..

[14] Valkonen M., Kuusi T. (1997). Spectrophotometric assay for total peroxyl radical-trapping antioxidant potential in human serum. J. Lipid Res..

[15] Pires M.A., Pastrana L.M., Fuciños P., Abreu C.S., Oliveira S.M. (2020). Sensorial perception of astringency: Oral mechanisms and current analysis methods. Foods.

[16] Van Hul M., Cani P.D. (2019). Targeting carbohydrates and polyphenols for a healthy microbiome and healthy weight. Curr. Nutr. Rep..

[17] Cirkovic Velickovic T.D., Stanic-Vucinic D.J. (2018). The role of dietary phenolic compounds in protein digestion and processing technologies to improve their antinutritive properties. Compr. Rev. Food Sci. Food Saf..

[18] Cai Z.-Y., Li X.-M., Liang J.-P., Xiang L.-P., Wang K.-R., Shi Y.-L., Yang R., Shi M., Ye J.-H., Lu J.-L. (2018). Bioavailability of tea catechins and its improvement. Molecules.

[19] Nishijima T., Iwai K., Saito Y., Takida Y., Matsue H. (2009). Chronic ingestion of apple pectin can enhance the absorption of quercetin. J. Agric. Food Chem..

[20] Zhu F. (2018). Interactions between cell wall polysaccharides and polyphenols. Crit. Rev. Food Sci. Nutr..

[21] Haider K., Wilde P., Kontogiorgos V. (2020). Digestion and metabolism of pectin. Pectin: Technological and Physiological Properties.

[22] Williamson G., Clifford M.N. (2017). Role of the small intestine, colon and microbiota in determining the metabolic fate of polyphenols. Biochem. Pharmacol..

[23] Paturi G., Butts C.A., Stoklosinski H., Herath T.D., Monro J.A. (2017). Short-Term feeding of fermentable dietary fibres influences the gut microbiota composition and metabolic activity in rats. Int. J. Food Sci. Technol..

[24] Donoso F., Egerton S., Bastiaanssen T.F.S., Fitzgerald P., Gite S., Fouhy F., Ross R.P., Stanton C., Dinan T.G., Cryan J.F. (2020). Polyphenols selectively reverse early-life stress-induced behavioural, neurochemical and microbiota changes in the rat. Psychoneuroendocrinology.

[25] Van de Wouw M., Boehme M., Lyte J.M., Wiley N., Strain C., O’Sullivan O., Clarke G., Stanton C., Dinan T.G., Cryan J.F. (2018). Short-chain fatty acids: Microbial metabolites that alleviate stress-induced brain–gut axis alterations. J. Physiol..

[26] Gulcin İ. (2020). Antioxidants and antioxidant methods: An updated overview. Arch. Toxicol..

[27] Pellegrini N., Serafini M., Colombi B., Del Rio D., Salvatore S., Bianchi M., Brighenti F. (2003). Total antioxidant capacity of plant foods, beverages and oils consumed in italy assessed by three different in vitro assays. J. Nutr..

[28] Denev P., Ciz M., Ambrozova G., Lojek A., Yanakieva I., Kratchanova M. (2010). Solid-phase extraction of berries’ anthocyanins and evaluation of their antioxidative properties. Food Chem..

[29] Mikami T., Sorimachi M. (2017). Uric acid contributes greatly to hepatic antioxidant capacity besides protein. Physiol. Res..

[30] Koguchi T., Tadokoro T. (2019). Beneficial effect of dietary fiber on hyperuricemia in rats and humans: A review. Int. J. Vitam. Nutr. Res..

