# Supplementary material for: Pectin Influences the Absorption and Metabolism of Polyphenols from Blackcurrant and Green Tea in Rats
Source: Foods. 2021 Apr 9;10(4):813. doi: 10.3390/foods10040813 (PMC8070010; doi:10.3390/foods10040813)
Supplement: Supplementary file 1 [file foods-10-00813-s001.pdf]

Supplementary data

Table S1. Relative peak areas of polyphenols in rat urine.

Precursor ion M-H, m/z	Product ion	Polyphenol	Control	Blackcurrant and green tea	Blackcurrant, green tea and pectin (4%)	Blackcurrant, green tea and pectin (8%)
		Catechins				
289	245(100), 205(30), 179(10)	Catechin	0	0	0	0
289	245(100), 205(30), 179(10)	Epicatechin	0	0	0	0
305	179(100), 221(90), 219(70), 261(45), 165(25), 125(20), 137(15), 287(10)	Gallocatechin	0	0	0	0
305	179(100), 221(90), 219(70), 261(45), 165(25), 125(20), 137(15), 287(10)	Epigallocatechin	0	0	0	0
441	289, 169(20), 331(20), 271(10)	Catechin gallate	0	0	0	0
441	289, 169(20), 331(20), 271(10)	Epicatechin gallate	0	0	0	0
457	169, 331(50), 305(25), 287(10), 269(7)	Gallocatechin gallate	0	0	0	0
457	169, 331(50), 305(25), 287(10), 269(7)	Epigallocatechin gallate	0	0	0	0
545	369(193)	Catechin diglucuronide sulfate	0	243	233	0
465	289(245, 205)	Epicatechin glucuronide	0	3263	3280	4352
479	303(259, 285, 244, 219)	Methyl epicatechin glucuronide	0	2384	2834	674
481	305(179, 221, 219, 261, 165)	Gallocatechin glucuronide	0	592	566	538
495	319(301, 137), 175, 477	Methyl gallocatechin glucuronide	0	1091	1357	399
683	507(331(316)), 331	Trimethyl catechin diglucuronide	0	137	123	114
		Anthocyanins				
449	287(213, 259, 231)	Cyanidin glucoside	0	408	130	258
595	287, 449	Cyanidin rutinoside	0	4508	2723	2071

465	303(257, 229, 247)	Delphinidin glucoside	0	117	115	0
611	303(257, 229, 247), 465	Delphinidin rutinoside	0	1133	516	650
479	317(302, 285, 261, 139)	Petunidin glucoside	0	328	247	73
625	317(302, 274, 257)	Petunidin rutinoside	0	508	343	217
609	301(286), 463	Peonidin rutinoside	0	2058	1418	772
477	301(286)	Methyl cyanidin glucuronide	0	326	214	0
669	317(302, 285), 493	Methyl delphinidin diglucuronide	0	1030	598	535
493	317(302, 285)	Methyl delphinidin glucuronide	0	239	265	0
		Flavonols	0	0	0	0
463	301(179, 151)	Quercetin glucoside	0	0	0	0
609	301, 463	Quercetin rutinoside	0	0	0	0
479	317(179, 151, 299, 302)	Myricetin glucoside	0	0	0	0
625	317, 479	Myricetin rutinoside	0	0	0	0
477	301(179, 151)	Quercetin glucuronide	0	0	0	0
491	315(300)	Methyl quercetin glucuronide	0	0	0	0
653	477(301(179, 151))	Quercetin diglucuronide	0	373	310	77
667	491, 315(300)	Methyl quercetin diglucuronide	0	1381	984	578
493	317(179, 151, 299, 302)	Myricetin glucuronide	0	114	76	0
669	493(317(179, 151, 299, 302))	Myricetin diglucuronide	0	557	559	38
829	653, 477, 301	Quercetin triglucuronide	0	387	351	0
637	461(285(257, 267, 241, 229, 151))	Kaempferol diglucuronide	0	379	306	323
845	669, 493, 317	Myricetin triglucuronide	0	931	678	272
		Phenolic acids				
153	109(65)	3,4 dihydroxybenzoic acid	0	1074	1167	1337
153	109(65)	2,4 dihydroxybenzoic acid	0	2535	4050	2967

Table S2. Relative peak areas of polyphenols in rat feces.

Precursor ion M-H, m/z	Product ion	Polyphenol	Control	Blackcurrant and green tea	Blackcurrant, green tea and pectin (4%)	Blackcurrant, green tea and pectin (8%)
		Catechins				
289	245(100), 205(30), 179(10)	Catechin	0	0	0	0
289	245(100), 205(30), 179(10)	Epicatechin	0	9186	15307	0
305	179(100), 221(90), 219(70), 261(45), 165(25), 125(20), 137(15), 287(10)	Gallocatechin	0	2698	6271	198
305	179(100), 221(90), 219(70), 261(45), 165(25), 125(20), 137(15), 287(10)	Epigallocatechin	0	45702	42972	4381
441	289, 169(20), 331(20), 271(10)	Catechin gallate	0	501	599	0
441	289, 169(20), 331(20), 271(10)	Epicatechin gallate	0	4348	3931	275
457	169, 331(50), 305(25), 287(10), 269(7)	Gallocatechin gallate	0	1855	1708	106
457	169, 331(50), 305(25), 287(10), 269(7)	Epigallocatechin gallate	0	7137	6318	407
545	369(193)	Catechin diglucuronide sulfate	0	0	0	0
465	289(245, 205)	Epicatechin glucuronide	0	0	0	0
479	303(259, 285, 244, 219)	Methyl epicatechin glucuronide	0	0	0	0
481	305(179, 221, 219, 261, 165)	Gallocatechin glucuronide	0	0	0	0
495	319(301, 137), 175, 477	Methyl gallocatechin glucuronide	0	0	0	0
683	507(331(316)), 331	Trimethyl catechin diglucuronide	0	0	0	0
		Anthocyanins	0	0	0	0
449	287(213, 259, 231)	Cyanidin glucoside	0	3323	200	217
595	287, 449	Cyanidin rutinoside	0	40965	1014	706
465	303(257, 229, 247)	Delphinidin glucoside	0	0	0	0

611	303(257, 229, 247), 465	Delphinidin rutinoside	0	46668	993	556
479	317(302, 285, 261, 139)	Petunidin glucoside	0	182	11	0
625	317(302, 274, 257)	Petunidin rutinoside	0	1927	0	0
609	301(286), 463	Peonidin rutinoside	0	2514	0	0
477	301(286)	Methyl cyanidin glucuronide	0	0	0	0
669	317(302, 285), 493	Methyl delphinidin diglucuronide	0	0	0	0
493	317(302, 285)	Methyl delphinidin glucuronide	0	0	0	0
		Flavonols	0	0	0	0
463	301(179, 151)	Quercetin glucoside	0	3823	986	409
609	301, 463	Quercetin rutinoside	0	0	0	0
479	317(179, 151, 299, 302)	Myricetin glucoside	0	12475	1642	560
625	317, 479	Myricetin rutinoside	0	0	0	0
477	301(179, 151)	Quercetin glucuronide	0	0	0	0
491	315(300)	Methyl quercetin glucuronide	0	0	0	0
653	477(301(179, 151))	Quercetin diglucuronide	0	0	0	0
667	491, 315(300)	Methyl quercetin diglucuronide	0	0	0	0
493	317(179, 151, 299, 302)	Myricetin glucuronide	0	0	0	0
669	493(317(179, 151, 299, 302))	Myricetin diglucuronide	0	0	0	0
829	653, 477, 301	Quercetin triglucuronide	0	0	0	0
637	461(285(257, 267, 241, 229, 151))	Kaempferol diglucuronide	0	0	0	0
845	669, 493, 317	Myricetin triglucuronide	0	0	0	0
		Phenolic acids	0	0	0	0
153	109(65)	3,4 dihydroxybenzoic acid	0	1973	3088	1098
153	109(65)	2,4 dihydroxybenzoic acid	0	3010	3251	2798

Table S3. Relative peak areas of polyphenols in rat plasma.

Precursor ion M-H, m/z	Product ion	Polyphenol	Control	Blackcurrant and green tea	Blackcurrant, green tea and pectin (4%)	Blackcurrant, green tea and pectin (8%)
		Catechins				
289	245(100), 205(30), 179(10)	Catechin	0	28	14	10
289	245(100), 205(30), 179(10)	Epicatechin	0	0	0	0
305	179(100), 221(90), 219(70), 261(45), 165(25), 125(20), 137(15), 287(10)	Gallocatechin	0	0	0	0
305	179(100), 221(90), 219(70), 261(45), 165(25), 125(20), 137(15), 287(10)	Epigallocatechin	0	0	0	0
441	289, 169(20), 331(20), 271(10)	Catechin gallate	0	0	0	0
441	289, 169(20), 331(20), 271(10)	Epicatechin gallate	0	0	0	0
457	169, 331(50), 305(25), 287(10), 269(7)	Gallocatechin gallate	0	0	0	0
457	169, 331(50), 305(25), 287(10), 269(7)	Epigallocatechin gallate	0	0	0	0
545	369(193)	Catechin diglucuronide sulfate	0	0	0	0
465	289(245, 205)	Epicatechin glucuronide	0	41	52	41
479	303(259, 285, 244, 219)	Methyl epicatechin glucuronide	0	64	92	62
481	305(179, 221, 219, 261, 165)	Gallocatechin glucuronide	0	3	0	0
495	319(301, 137), 175, 477	Methyl gallocatechin glucuronide	0	19	21	19
683	507(331(316)), 331	Trimethyl catechin diglucuronide	0	86	138	64
		Anthocyanins	0	0	0	0
449	287(213, 259, 231)	Cyanidin glucoside	0	0	0	0
595	287, 449	Cyanidin rutinoside	0	0	0	0
465	303(257, 229, 247)	Delphinidin glucoside	0	0	0	0
611	303(257, 229, 247), 465	Delphinidin rutinoside	0	0	0	0

479	317(302, 285, 261, 139)	Petunidin glucoside	0	0	0	0
625	317(302, 274, 257)	Petunidin rutinoside	0	0	0	0
609	301(286), 463	Peonidin rutinoside	0	0	0	0
477	301(286)	Methyl cyanidin glucuronide	0	0	0	0
669	317(302, 285), 493	Methyl delphinidin diglucuronide	0	0	0	0
493	317(302, 285)	Methyl delphinidin glucuronide	0	0	0	0
		Flavonols	0	0	0	0
463	301(179, 151)	Quercetin glucoside	0	0	0	0
609	301, 463	Quercetin rutinoside	0	16	25	23
479	317(179, 151, 299, 302)	Myricetin glucoside	0	0	0	0
625	317, 479	Myricetin rutinoside	0	0	0	0
477	301(179, 151)	Quercetin glucuronide	0	38	50	48
491	315(300)	Methyl quercetin glucuronide	0	25	25	15
653	477(301(179, 151))	Quercetin diglucuronide	0	161	157	159
667	491, 315(300)	Methyl quercetin diglucuronide	0	56	65	35
493	317(179, 151, 299, 302)	Myricetin glucuronide	0	64	53	54
669	493(317(179, 151, 299, 302))	Myricetin diglucuronide	0	97	100	84
829	653, 477, 301	Quercetin triglucuronide	0	0	0	0
637	461(285(257, 267, 241, 229, 151))	Kaempferol diglucuronide	0	0	0	0
845	669, 493, 317	Myricetin triglucuronide	0	0	0	0
		Phenolic acids	0	0	0	0
153	109(65)	3,4 dihydroxybenzoic acid	0	0	0	0
153	109(65)	2,4 dihydroxybenzoic acid	0	0	0	0
